# Evaluating the effectiveness of AI-enhanced “One Body, Two Wings” pharmacovigilance models in China: a nationwide survey on medication safety and risk management

**DOI:** 10.3389/frhs.2025.1655726

**Published:** 2025-10-08

**Authors:** Jian Yang, Yi Sun, Fan Li, Quanzhi Wei, Jincui Wei, Yingxiong Zhang

**Affiliations:** ^1^ School of Pharmaceutical Sciences & Yunnan Provincial Key Laboratory of Pharmacology for Natural Products, Kunming Medical University, Kunming, Yunnan, China; ^2^ Yunnan Provincial Center for Drug Policy Research, Kunming, Yunnan, China; ^3^ Yuxi Drug Evaluation Center (Yuxi Adverse Drug Reaction Monitoring Center), Yuxi, Yunnan, China; ^4^ Xinping Yi and Dai Autonomous County Drug Safety Monitoring and Evaluation Center, Yuxi, Yunnan, China; ^5^ Sailing Pharmaceutical Technology Group Co., Ltd., Yuxi, Yunnan, China

**Keywords:** pharmacovigilance, artificial intelligence, "One body, Two Wings" model, medication safety, risk management

## Abstract

**Background:**

This study evaluates the effectiveness of AI-enhanced “One Body Two Wings” pharmacovigilance models in China, focusing on improving medication safety and risk management. As the pharmaceutical landscape grows more complex, integrating AI into pharmacovigilance offers the potential to enhance adverse drug reaction (ADR) detection and monitoring.

**Methods:**

A nationwide cross-sectional survey was conducted from June 25 to August 10, 2024, involving 1,000 participants from pharmacovigilance centers, hospitals, corporations, and the general public. Participants were recruited through stratified convenience sampling to ensure a broad geographical and professional representation. Data were collected through a validated questionnaire and analyzed using ANOVA, regression analysis, decision tree models, and random forest algorithms. To ensure the validity of the predictive models, resampling (SMOTE) and class weighting techniques were employed to address significant class imbalance in the outcome variable.

**Results:**

The survey revealed that 43% of participants were hospital staff and 46% had more than 10 years of experience, with these expert groups expressing strong support for AI's role. Path analysis indicated that AI's effectiveness in processing ADR reports was strongly related to enhanced monitoring capabilities (standardized path coefficient: 0.85). Furthermore, logistic regression identified the perceived effectiveness of information systems as a significant predictor of positive attitudes toward the model (odds ratio: 1.703). Crucially, a random forest model, adjusted for class imbalance, confirmed that information systems effectiveness was the most significant predictor of the model's success (mean importance: 0.53 ± 0.05), achieving robust performance with a weighted F1-score of 0.94 and an AUC-ROC of 0.89.

**Conclusion:**

The findings confirm AI's potential to enhance pharmacovigilance, especially in ADR monitoring. However, the study concludes that successful AI integration is predicated on a robust information systems infrastructure, which the data identified as the most critical foundational element. Therefore, optimizing pharmacovigilance in China requires prioritized investment in both this foundational IT and supportive organizational frameworks.

## 1 Introduction

Pharmacovigilance, the science and activities relating to the detection, assessment, understanding, and prevention of adverse effects or any other drug-related problems, is a cornerstone of drug safety (1). As the pharmaceutical industry continues to innovate with new drugs, the associated risks also grow, necessitating robust pharmacovigilance systems. In response, China has implemented several strategic initiatives to enhance its pharmacovigilance capabilities. The introduction of the Marketing Authorization Holder (MAH) system in 2015 was a significant step towards aligning China's drug regulation with international standards (2). This system places the responsibility for drug safety squarely on the MAH, which includes pharmaceutical companies and research institutions that have obtained drug registration certificates (3). In 2020, the National Medical Products Administration (NMPA) introduced the “One Body, Two Wings” model to further strengthen China's pharmacovigilance framework. This model positions pharmacovigilance institutions as the central body, with marketing authorization holders and healthcare institutions serving as the supporting wings. This structure aims to enhance the monitoring and evaluation of adverse drug reactions (ADRs) across the nation, ensuring a systematic and collaborative approach to drug safety (4).

The importance of pharmacovigilance has been further underscored by global trends, where the rapid introduction of new drugs can outpace the capacity of existing monitoring systems (5). For example, studies in Africa have highlighted resource limitations as a major challenge, requiring innovative solutions to ensure drug safety (6). In parallel, global pharmacovigilance strategies increasingly emphasize the role of data-driven approaches, from Europe's use of statistical modeling for risk assessment to the international consensus on the potential of AI to enhance safety reporting (7).

In China, this has led to a national push to integrate pharmacovigilance practices more deeply into the healthcare system. However, despite these efforts, significant challenges remain. These include inadequate monitoring mechanisms, insufficient communication between stakeholders, and delays in responding to medication risks. These issues are particularly pronounced at the regional level, where the implementation of pharmacovigilance systems can be inconsistent (8). Recent studies provide a snapshot of the current state of pharmacovigilance in China, revealing both progress and persistent challenges. For example, a survey of MAHs in Guangzhou showed that while basic pharmacovigilance systems have been established, there are still major deficiencies in organizational structures, ADR reporting capabilities, and risk management processes (9). Similarly, research in Hainan indicates that a significant proportion of MAHs lack dedicated pharmacovigilance departments and full-time staff, which are critical for effective monitoring and management of drug safety issues (10).

The global trend towards the integration of Artificial Intelligence (AI) in pharmacovigilance has also begun to influence practices in China. AI technologies offer the potential to enhance the detection and reporting of ADRs by analyzing vast datasets from electronic health records, social media, and other sources (11). These tools can identify safety signals faster and more accurately than traditional methods. However, the implementation of AI in pharmacovigilance is still nascent, with challenges such as data quality, regulatory hurdles, and the need for specialized personnel slowing down adoption (12). Moreover, a lack of comprehensive studies evaluating the effectiveness of AI-driven pharmacovigilance systems on a national scale persists (13).

Despite advancements in pharmacovigilance practices and the integration of AI, significant research gaps remain. Most existing studies are region-specific or focus on individual institutions, leaving a gap in understanding the effectiveness of these systems across different regions of China (14). Additionally, while AI shows promise in enhancing pharmacovigilance, its integration into the existing “One Body, Two Wings” pharmacovigilance model has not been thoroughly explored. The potential for AI to improve the speed and accuracy of ADR reporting and thereby enhance medication safety and risk management remains an under-researched area (15).

This study aims to bridge the knowledge gap by comprehensively evaluating the effectiveness of AI-enhanced “One Body, Two Wings” pharmacovigilance models across China. We seek to understand the current issues in pharmacovigilance management, assess the levels of familiarity and perceived effectiveness of the “One Body, Two Wings” model, and explore how AI can enhance these processes. Our goal is to provide insights that will help tailor pharmacovigilance strategies to the needs of various stakeholders, including pharmacovigilance center managers, hospital staff, corporate employees, and the general public. By examining the knowledge, attitudes, and practices related to these models, this research will contribute to the evidence base needed to refine pharmacovigilance policies and interventions, ensuring they are not only well-conceived but also effectively implemented across the healthcare system.

## 2 Methods

### 2.1 Study design and setting

This cross-sectional study, conducted across China from June 25 to August 10, 2024, evaluated the effectiveness of AI-enhanced “One Body, Two Wings” pharmacovigilance models and explored medication safety and risk management practices. The study utilized a quantitative research design and employed the “Questionnaire Star platform”, a widely-used online tool in China, for data collection. This approach aimed to capture the perceptions and practices of stakeholders, including pharmacovigilance center managers, hospital staff, corporate employees, and the general public, regarding the pharmacovigilance framework and AI's role in enhancing it. The study began with a pilot phase involving 50 questionnaires collected via convenience sampling to refine the survey design. The main survey phase then gathered a diverse set of responses, providing valuable insights into pharmacovigilance management, familiarity with the “One Body, Two Wings” model, and the perceived effectiveness of AI across different regions of China. These findings contribute to understanding and improving the national pharmacovigilance system.

### 2.2 Participants and data collection

This quantitative cross-sectional study targeted a diverse range of stakeholders across China, including pharmacovigilance center managers, hospital staff, corporate employees, and the public, all aged 18 and older. The inclusion criteria were specifically defined to capture perspectives from key professional groups involved in pharmacovigilance, and the recruitment process was an active selection strategy. Participants were selected from various regions to ensure a representative sample reflecting different perspectives on the “One Body, Two Wings” pharmacovigilance model and AI's role in enhancing these processes. Recruitment was conducted using a stratified convenience and snowball sampling approach. Initial survey links were distributed to a network of key contacts in different professional groups and diverse geographical regions (e.g., Shanghai, Yunnan, Hebei, Guangdong, Hunan, and Jiangxi) who were then encouraged to share the survey within their respective networks. This method facilitated a broad reach across different provinces. The survey began with informed consent, ensuring participants were fully aware of the study's objectives. Distributed via the “Questionnaire Star platform”, the questionnaire included Likert scale and multiple-choice questions to assess knowledge, attitudes, and practices regarding pharmacovigilance, the “One Body, Two Wings” model, and AI integration. While this online convenience sampling method may be subject to selection bias (e.g., favoring more technologically proficient participants), it was deemed the most effective approach for achieving a broad, nationwide geographic and multi-stakeholder reach for this exploratory study. The focus of the analysis is on the relationships between variables rather than estimating precise population prevalence. The questionnaire, specifically developed for this study, was reviewed and validated by experts in pharmacovigilance and survey design. To further ensure reliability, a pilot study was conducted on 50 participants, and the questionnaire's internal consistency was assessed using Cronbach's alpha, which yielded a coefficient of 0.82, indicating good reliability. The sample size was calculated using the formula: *z^2^ × p* *×* *(1−p)/d^2^*, where “z” is the z-score for a 95% confidence level (1.96), “*p*” is the estimated proportion of an attribute in the population (set at 0.5), and “d” is the margin of error (0.04). This resulted in an initial sample size of 601. To ensure data integrity, criteria for valid responses included unique submissions (verified by IP addresses), a minimum completion time of 7 min, and correct answers to two logical consistency questions. Out of 1,067 responses, 1,000 valid responses were retained after excluding invalid entries. This final sample size ensured statistical significance, providing a robust dataset for analyzing the effectiveness and perceptions of the AI-enhanced “One Body, Two Wings” pharmacovigilance model across China.

### 2.3 Data analysis

Data preprocessing and analysis were performed using IBM SPSS Statistics (version 22.0) and Python (version 3.10). Descriptive statistics summarized participant demographics. ANOVA and regression analysis identified significant patterns and predictive relationships. Exploratory Factor Analysis (EFA) uncovered underlying factor structures, and path analysis modeled causal relationships. Advanced data mining techniques, such as decision tree and random forest algorithms, were used to predict key factors influencing the perceived effectiveness of the management model. To address significant class imbalance in the outcome variable, the Synthetic Minority Over-sampling Technique (SMOTE) and class weighting adjustments were respectively applied to the training data for the tree-based models. Performance was evaluated using balanced metrics, including the weighted F1-score and AUC-ROC. All statistical analyses used a two-tailed test with a *p*-value < 0.05 considered statistically significant.

### 2.4 Ethical considerations

This study adhered to ethical standards and received approval from Kunming Medical University's Institutional Review Board (Protocol Number: 2024003157). All methods were performed in accordance with relevant guidelines and regulations, including the Declaration of Helsinki. Informed consent was obtained from all participants, ensuring they were fully aware of the study's purpose, procedures, and their confidentiality rights. To protect participant privacy, all identifying information, including names and other identifiers, has been removed from the manuscript and Supplementary Materials. No unauthorized identifying images or videos were used.

## 3 Results

### 3.1 Comprehensive analysis of respondent characteristics and model familiarity

This analysis provides a comprehensive overview of key demographic characteristics of the survey respondents and their familiarity with the integrated One Body, Two Wings coordinated pharmacovigilance management model. Table 1 indicates that the majority of participants are within the 26–35 age group, reflecting a significant involvement of younger professionals in the field of pharmacovigilance. The survey is predominantly represented by hospital staff and corporate employees, suggesting that these sectors are the most actively engaged in pharmacovigilance initiatives. Notably, 46% of the respondents have more than 10 years of work experience, highlighting the presence of a highly experienced cohort among the participants. Gender distribution shows a higher participation rate among females, who make up 78% of the respondents. The survey's nationwide scope is evident from its broad geographical representation, with participants from all regions of China, including the east (Shanghai), west (Yunnan), north (Hebei), south (Guangdong), and central regions (Hunan and Jiangxi). This diverse geographical coverage reinforces the national relevance of the survey results, underscoring the engagement of a well-experienced professional group in pharmacovigilance activities.

**Table 1:** Demographic distribution and model familiarity among survey respondents.

Item	Distribution	Proportion
Your role		
Pharmacovigilance Center Manager	73	7%
Hospital Staff	428	43%
Corporate Employee	372	37%
Public	127	13%
Your age		
18–25	107	11%
26–35	385	39%
36–45	328	33%
46–60	173	17%
Above 60	7	1%
Your gender		
Male	220	22%
Female	780	78%
Years of work experience		
1–3 years	204	20%
4–7 years	207	21%
8–10 years	134	13%
More than 10 years	455	46%

### 3.2 Analysis of AI capabilities in pharmacovigilance across different roles

**Figure 1:**
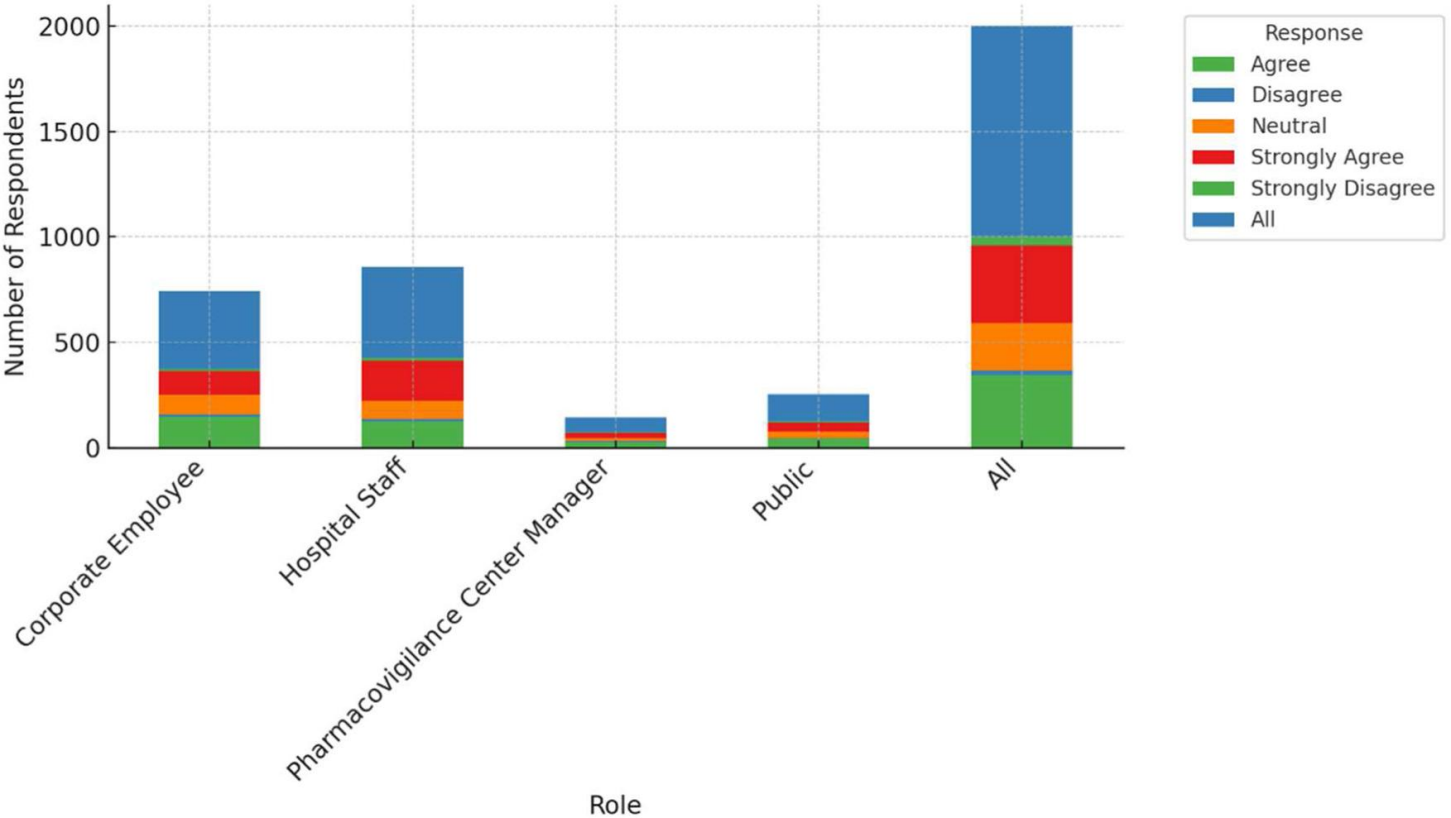
Agreement with AI capabilities in pharmacovigilance by role.

This study examines the perceptions of AI's role in enhancing pharmacovigilance across various professional groups, including corporate employees, hospital staff, pharmacovigilance center managers, and the general public. As shown in Figure 1, there are notable differences in agreement levels regarding AI's effectiveness in monitoring and warning systems. A significant proportion of pharmacovigilance center managers (around 40%) strongly agree that AI can enhance these systems, with most holding positive views. Corporate employees also show strong support, with 147 out of 372 agreeing with AI's potential. However, responses from hospital staff are more mixed, with 126 out of 428 agreeing, but a considerable number expressing skepticism. The general public's opinions are divided as well, with 42 out of 127 agreeing and 11 disagreeing, reflecting varied familiarity with AI technologies. Table 2 provides a detailed cross-tabulation of these responses, highlighting the diversity in perceptions across different roles.

**Table 2:** Cross-tabulation of agreement with AI capabilities in pharmacovigilance by role.

Your role	Agree	Disagree	Neutral	Strongly agree	Strongly disagree	All
Corporate Employee	147	11	91	113	10	372
Hospital Staff	126	10	85	190	17	428
Pharmacovigilance Center Manager	29	1	14	26	3	73
Public	42	2	33	41	9	127
All	344	24	223	370	39	1,000

### 3.3 Group differences in AI attitudes in pharmacovigilance: an ANOVA analysis

A one-way Analysis of Variance (ANOVA) was conducted to examine whether there are significant differences in attitudes towards Artificial Intelligence (AI) in pharmacovigilance among different professional groups, including Corporate Employees, Hospital Staff, Pharmacovigilance Center Managers, and the General Public. The AI attitude was measured on a Likert scale from 1 (Strongly Disagree) to 5 (Strongly Agree). Table 3 revealed a statistically significant difference in AI attitudes across these groups, with an F-statistic of 2.891 and a *p*-value of 0.034. This indicates that at least one group's mean attitude towards AI differs significantly from the others. While the analysis confirms that role-based differences exist, it does not specify which groups differ from each other; further *post-hoc* testing would be necessary to pinpoint these differences.

**Table 3:** ANOVA results for AI attitudes in pharmacovigilance.

Source	Sum of squares	Degrees of freedom	Mean square	F-value	*p*-value
Between Groups	2.891056	3	0.963685	2.891056	0.034
Within Groups	996.1089	996	1.000109		
Total	999	999			

### 3.4 Correlation analysis of pharmacovigilance management issues and monitoring system evaluation

**Figure 2:**
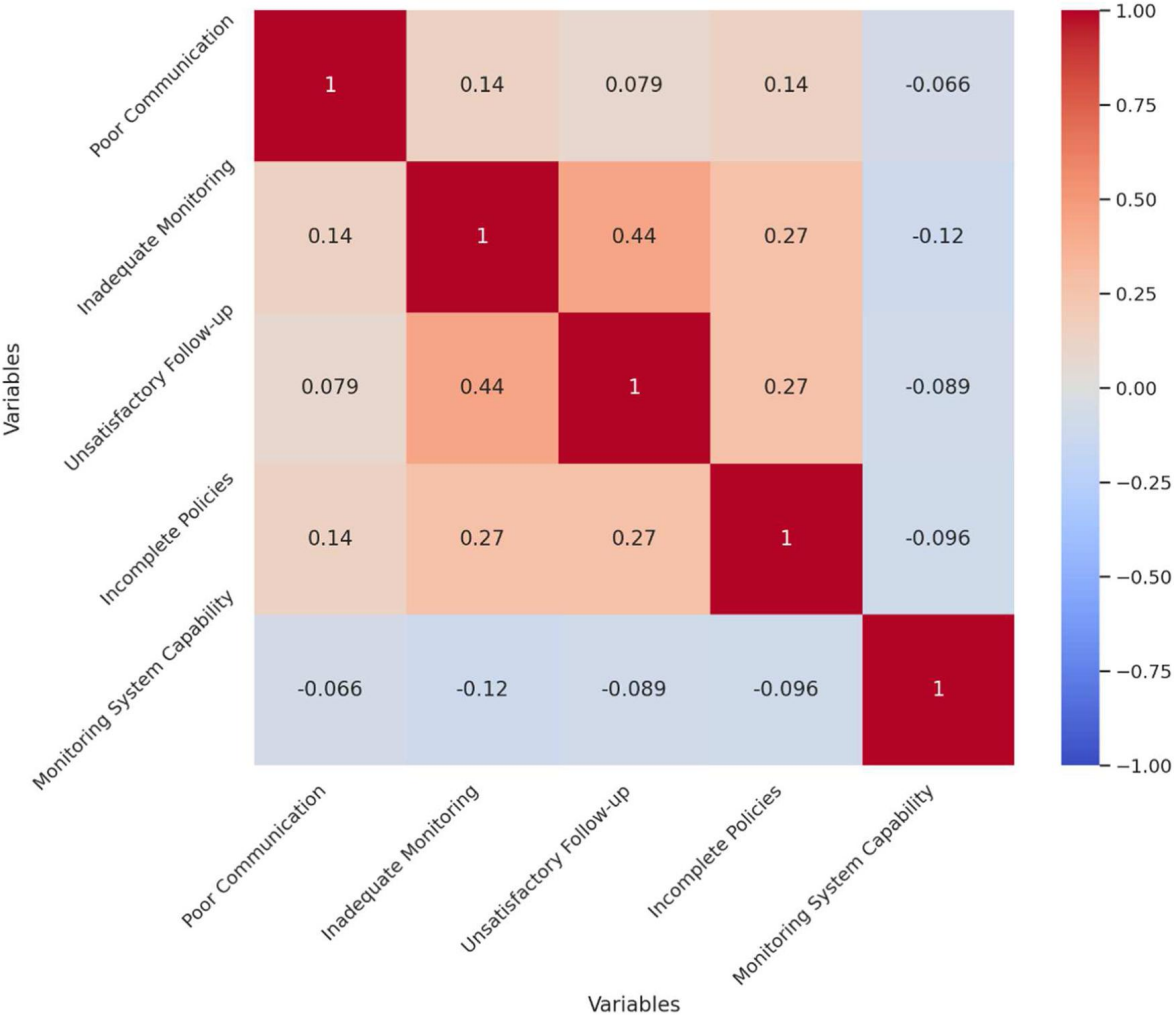
Correlation matrix of pharmacovigilance management issues and monitoring system evaluation.

The correlation analysis of the survey data reveals significant relationships between various aspects of pharmacovigilance management and the perceived effectiveness of the monitoring system. The analysis highlights that inadequate monitoring and warning mechanisms are moderately positively correlated with unsatisfactory follow-up measures (*r* = 0.44), suggesting that deficiencies in one area often coincide with issues in the other. This indicates that improving monitoring mechanisms could potentially enhance the follow-up procedures. Additionally, there is a smaller positive correlation between poor communication and inadequate monitoring mechanisms (*r* = 0.14), implying that communication challenges may exacerbate monitoring inadequacies, which underscores the need for better communication strategies to support monitoring efforts. Interestingly, the belief in the monitoring system's capability to detect potential medication risks shows a slight negative correlation with identified issues such as poor communication (*r* = −0.07), inadequate monitoring (*r* = −0.12), and incomplete policies (*r* = −0.10). This suggests that individuals who perceive significant issues in these areas tend to have lower confidence in the monitoring system's effectiveness. Figure 2 illustrates these correlations with a clear distinction between positive and negative relationships.

### 3.5 Factors influencing attitudes towards AI in pharmacovigilance: a multiple regression analysis

**Figure 3:**
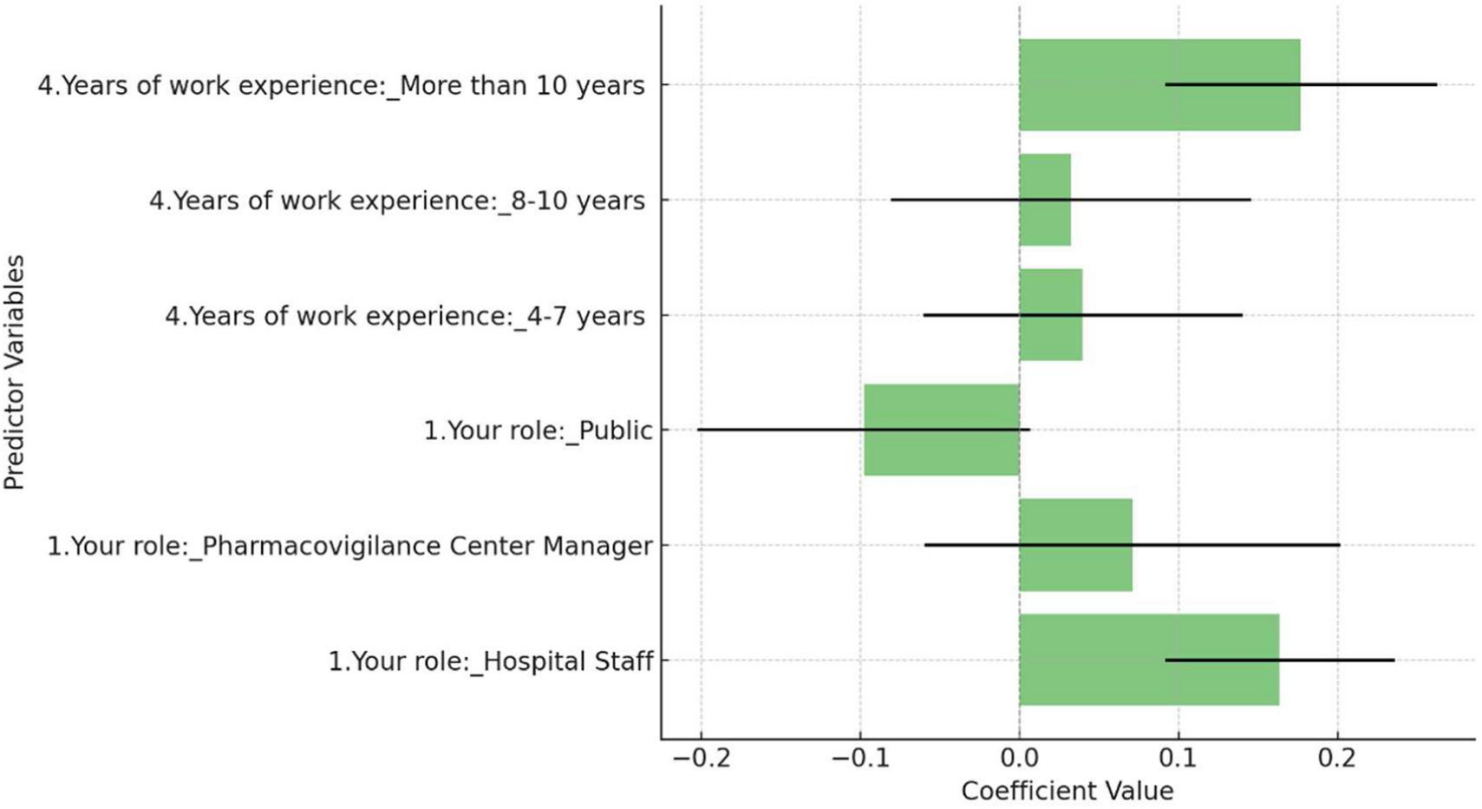
Regression coefficients for AI attitude in pharmacovigilance.

This study employed a multiple regression analysis to identify the factors influencing attitudes towards the use of Artificial Intelligence (AI) in pharmacovigilance. The analysis considered key variables including the respondent's role (categorized as Corporate Employee, Hospital Staff, Pharmacovigilance Center Manager, and Public) and years of work experience (grouped into 1–3 years, 4–7 years, 8–10 years, and more than 10 years). The dependent variable was the AI attitude score, measured on a Likert scale from 1 (Strongly Disagree) to 5 (Strongly Agree). Figure 3 revealed that Hospital Staff were significantly more likely to have a positive attitude towards AI in pharmacovigilance compared to the reference group, Corporate Employees, with a coefficient of 0.164 (*p* = 0.023). Additionally, respondents with more than 10 years of work experience showed a significant positive attitude towards AI, with a coefficient of 0.177 (*p* = 0.039), suggesting that greater professional experience correlates with a more favorable view of AI's effectiveness. The overall model was statistically significant (F-statistic = 2.421, *p* = 0.0250), though it explained only a small portion of the variance in attitudes (R-squared = 0.014), indicating that other unmeasured factors likely play a role.

### 3.6 Influence of AI-related beliefs on perceived effectiveness of pharmacovigilance models: a logistic regression analysis

**Figure 4:**
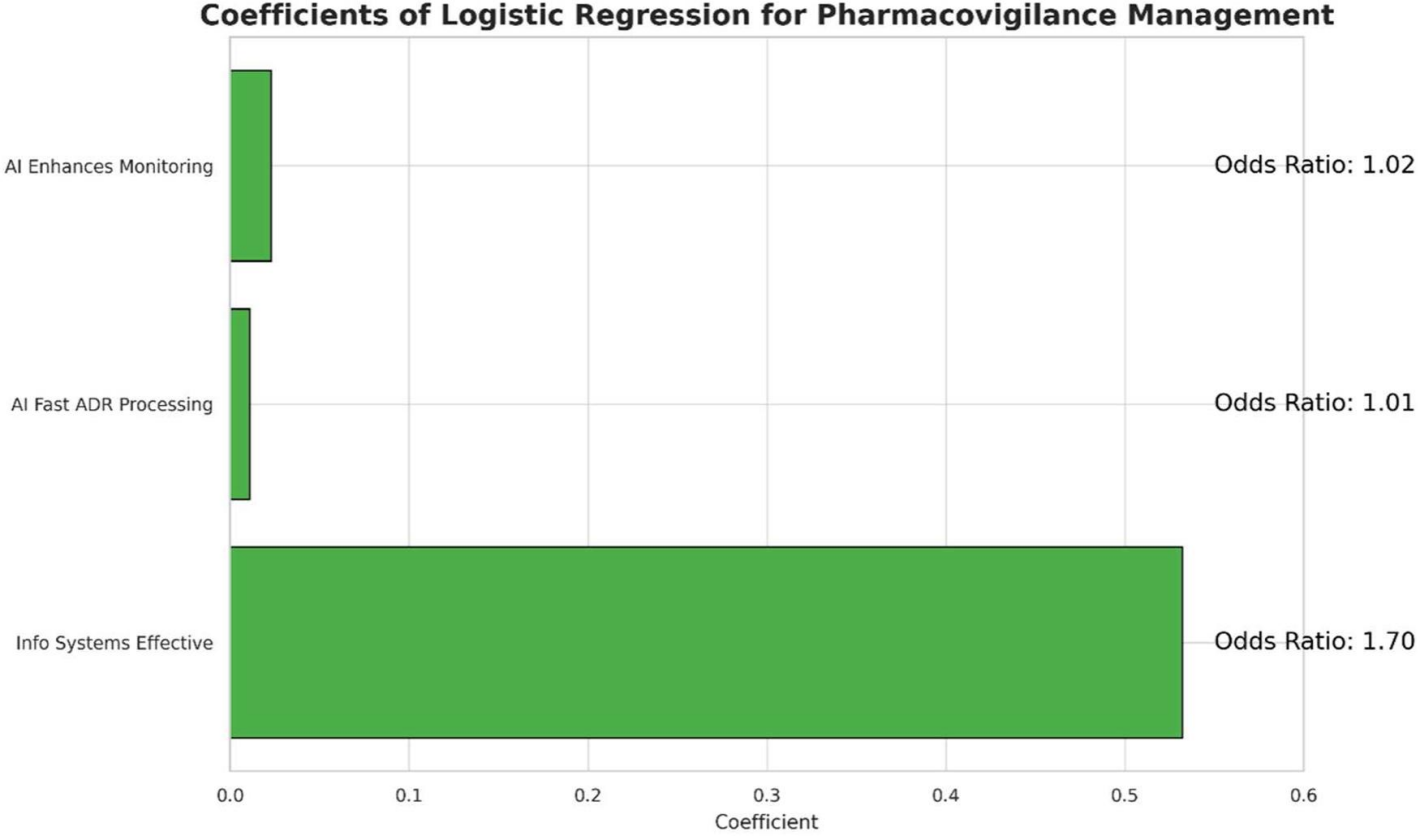
Coefficient plot for logistic regression analysis.

A logistic regression analysis was conducted to examine the association between specific beliefs regarding technology and the perceived effectiveness of the pharmacovigilance management model. The results are detailed in Table 4. The analysis identified the belief in the effectiveness of information systems as a statistically significant predictor; a higher rating on this factor increased the odds of a respondent perceiving the overall model as effective [OR = 1.703, 95% CI (1.482, 1.956), *p* < 0.001]. In contrast, neither the belief in AI's ability to enhance monitoring [OR = 1.023, 95% CI (0.920, 1.138), *p* = 0.670] nor its role in faster adverse drug reaction (ADR) processing [OR = 1.011, 95% CI (0.903, 1.131), *p* = 0.850] showed a statistically significant association with the outcome. These findings suggest that a robust information system is the primary factor associated with a positive perception of the management model's effectiveness (Figure 4).

**Table 4:** Logistic regression analysis of factors influencing perceived model effectiveness.

Predictor variable	Coefficient (β)	Std. Error	*p*-value	Odds ratio	95% CI for odds ratio
Info Systems Effective	0.532	0.071	<0.001	1.703	[1.482, 1.956]
AI Enhances Monitoring	0.023	0.054	0.670	1.023	[0.920, 1.138]
AI Fast ADR Processing	0.011	0.058	0.850	1.011	[0.903, 1.131]

### 3.7 Interpretation of factor loadings in pharmacovigilance management

**Figure 5:**
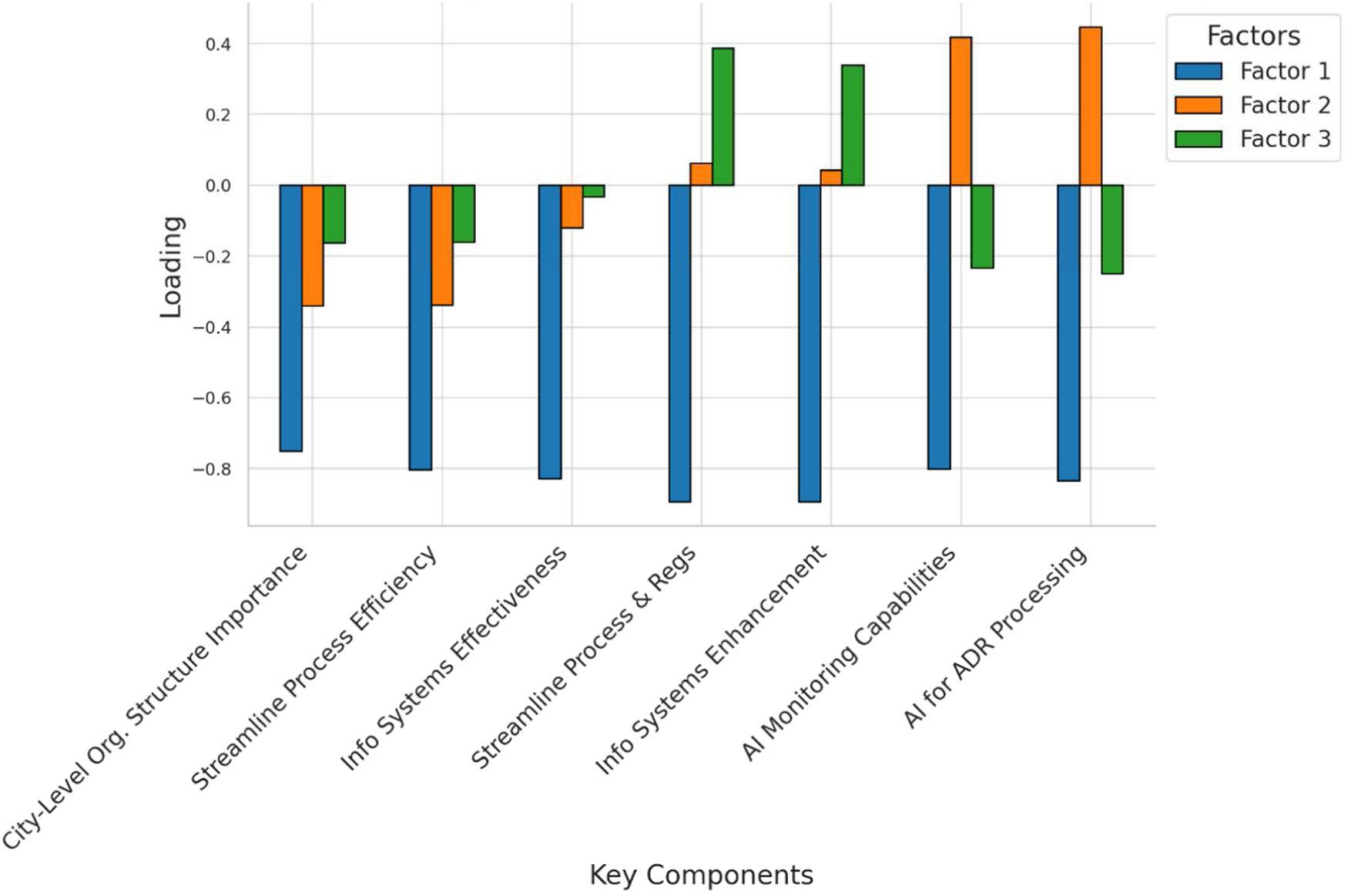
Factor loadings for key components in pharmacovigilance management.

The factor loadings analysis reveals three key components of effective pharmacovigilance management (Figure 5). The first factor is closely linked to organizational structure and process efficiency, with strong loadings on the importance of a city-level organizational structure (loading = 0.75) and streamlined process management (loading = 0.80). These findings highlight the value placed on efficient organizational frameworks. The second factor emphasizes technological integration, particularly artificial intelligence (AI), with notable loadings on AI's capability to enhance monitoring (loading = 0.80) and expedite adverse drug reaction (ADR) report processing (loading = 0.83). This underscores AI's transformative potential in pharmacovigilance. The third factor highlights the essential role of information systems in coordinated management, with a high loading on the belief that these systems significantly improve effectiveness (loading = 0.89). Overall, these results underscore the importance of both organizational and technological advancements, especially the integration of AI and information systems, in optimizing pharmacovigilance management.

### 3.8 Exploratory factor analysis of AI and information systems in pharmacovigilance

**Figure 6:**
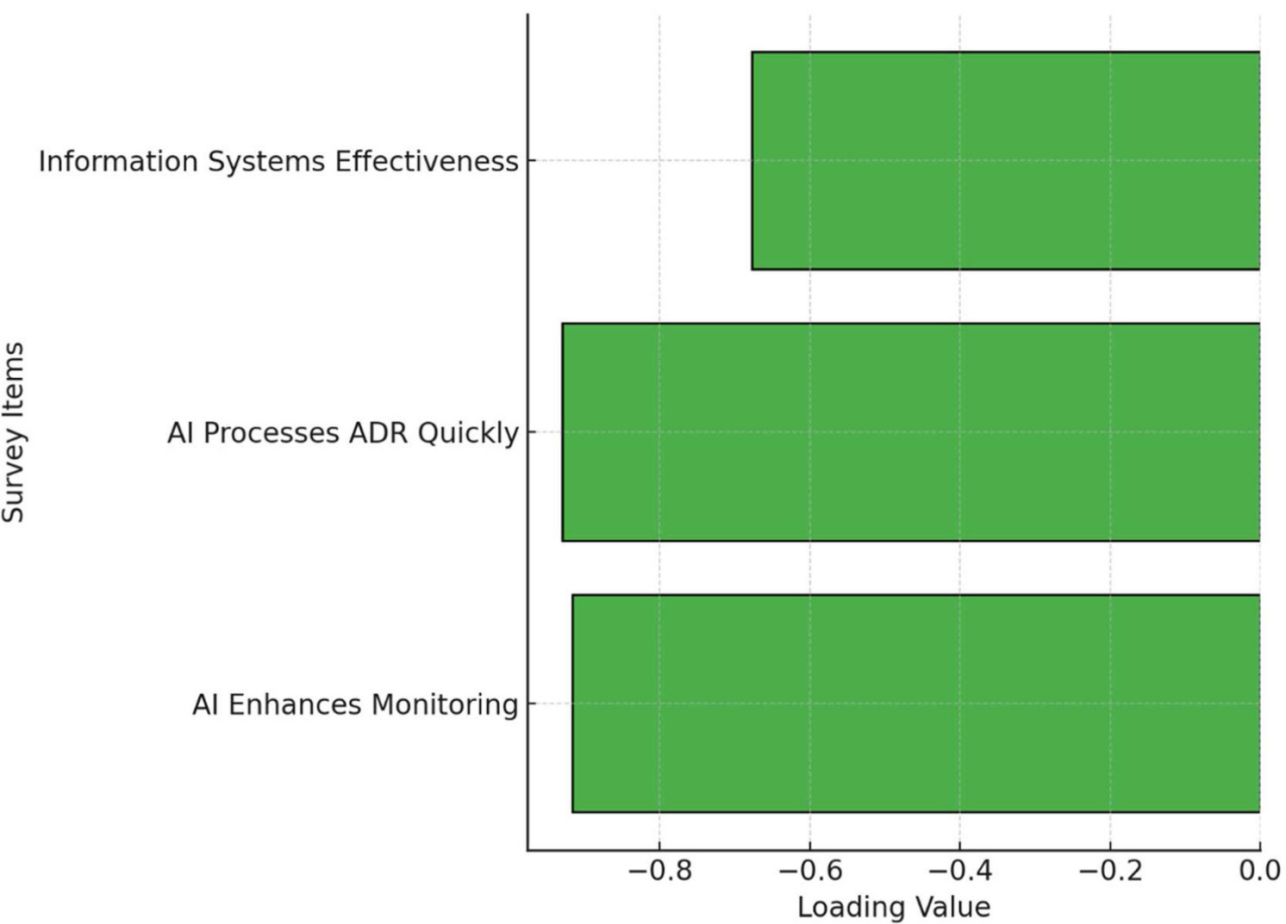
Factor loadings from exploratory factor analysis.

This study conducted an Exploratory Factor Analysis (EFA) to examine the underlying factor structure of survey items related to the application of Artificial Intelligence (AI) and information systems in pharmacovigilance. The analysis identified a single factor that captured the common variance among three key items. Specifically, the loading for the item “AI Enhances Monitoring” was −0.916, while “AI Processes ADR Quickly” had a loading of −0.930, and “Info Systems Enhance Effectiveness” showed a loading of −0.678. These results suggest that respondents strongly associate AI capabilities with the overall effectiveness of pharmacovigilance practices, with information systems also contributing significantly to this perception. The high factor loadings indicate that these items collectively measure a common underlying construct, likely representing confidence in the use of AI and information systems to improve pharmacovigilance. Figure 6 is a simplified visualization of the EFA results. For clarity, it presents the loadings in a horizontal bar chart format rather than a more complex matrix or network diagram, aiming to make the findings more accessible to a broader audience.

### 3.9 Impact of AI on pharmacovigilance: path analysis of monitoring, ADR processing, and information systems

**Figure 7:**
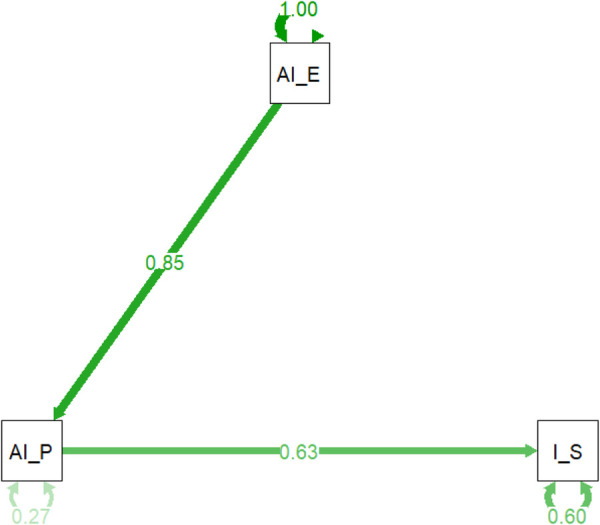
Path diagram of AI impact on pharmacovigilance management.

The path analysis model (Figure 7 and Table 5) reveals the intricate relationships between key variables in AI-enhanced pharmacovigilance. It confirms that AI's effectiveness in processing adverse drug reaction (ADR) reports (AI_P) significantly influences both the perceived enhancement of monitoring and warning capabilities (AI_E) and the overall effectiveness of information systems (I_S). The standardized path coefficient from AI_P to AI_E is 0.85, indicating a strong positive relationship, while the path from AI_P to I_S has a coefficient of 0.63, suggesting that effective AI in ADR processing directly contributes to perceived information system effectiveness. The model explains 27% of the variance in AI's processing capabilities and 60% of the variance in information system effectiveness, highlighting its robustness. These findings emphasize AI's pivotal role in enhancing pharmacovigilance, not only by improving specific tasks like ADR processing but also by strengthening the broader information systems framework. The significant path coefficients and high explained variance validate the model, offering strong evidence that integrating AI into pharmacovigilance enhances safety management systems.

**Table 5:** Standardized path coefficients and explained variance for AI impact on pharmacovigilance systems.

lhs	op	rhs	est.std	se	z	*p*_value	ci.lower	ci.upper
AI_Processes_ADR_Quickly	∼	AI_Enhances_Monitoring	0.851762	0.006929	122.9185	0	0.83818	0.865343
Information_Systems_Effectiveness	∼	AI_Processes_ADR_Quickly	0.631941	0.017968	35.16957	0	0.596724	0.667158
AI_Processes_ADR_Quickly	∼∼	AI_Processes_ADR_Quickly	0.274502	0.011805	23.25395	0	0.251366	0.297639
Information_Systems_Effectiveness	∼∼	Information_Systems_Effectiveness	0.60065	0.02271	26.44879	0	0.55614	0.645161
AI_Enhances_Monitoring	∼∼	AI_Enhances_Monitoring	1	0	NA	NA	1	1

This table provides the standardized estimates of the direct effects among the variables in the path model. The estimates indicate how strongly each independent variable predicts the dependent variable.

### 3.10 Analysis of decision tree results for pharmacovigilance management effectiveness

A decision tree model was constructed to identify predictors of pharmacovigilance management effectiveness. To ensure robustness against class imbalance, the Synthetic Minority Over-sampling Technique (SMOTE) was applied to the training data. The resulting model demonstrated strong predictive performance, achieving an overall accuracy of 92.1%, a weighted F1-score of 0.94, and a Cohen's Kappa of 0.58 (Table 6). Crucially, the model successfully identified the minority “Ineffective” class with a Recall of 0.65. Analysis of the pruned tree (maximum depth = 5) revealed that “Info Systems Effective” was the primary splitting feature, indicating its central role in predicting management effectiveness, with key classification rules detailed in Table 7.

**Table 6:** Classification report for decision tree model after SMOTE.

Class	Precision	Recall	F1-Score	Support
Ineffective	0.55	0.65	0.60	20
Effective	0.98	0.94	0.96	280
Accuracy			0.92	300
Macro Avg	0.77	0.80	0.78	300
Weighted Avg	0.95	0.92	0.94	300
Cohen's Kappa	0.58			

Cohen's Kappa = 0.58.

**Table 7:** Key decision rules for predicting “‘Ineffective” management from the decision tree model.

Rule No.	Condition 1	Condition 2	Condition 3	Predicted outcome
1	Info Systems Effective > 2.5	AI Enhances Monitoring > 1.5	Info Systems Effective ≤ 3.5	Ineffective
2	Info Systems Effective ≤ 2.5	AI Fast ADR Processing > 3.5	AI Enhances Monitoring ≤ 3.5	Ineffective

### 3.11 Feature importance analysis of random forest model in predicting pharmacovigilance management effectiveness

The random forest model was used to predict the effectiveness of the integrated One Body, Two Wings pharmacovigilance management model. To account for significant class imbalance, a class weighting strategy was implemented during model training. The resulting model produced reliable performance metrics, achieving an overall accuracy of 93.2%, a weighted F1-score of 0.94, and an AUC-ROC of 0.89 (Table 8). This balanced approach enabled the model to successfully classify the “Not Effective” category (Recall = 0.68). Feature importance analysis, stabilized via 10-fold cross-validation and calculated using the mean decrease in Gini impurity, revealed that the perceived effectiveness of information systems was the most influential factor (mean importance: 0.53 ± 0.05), followed by AI's role in enhancing monitoring (mean importance: 0.25 ± 0.03) and accelerating adverse drug reaction (ADR) processing (mean importance: 0.22 ± 0.03). These robust findings emphasize the critical role of information systems in shaping perceptions of the model's effectiveness.

**Table 8:** Classification report for random forest model with class weighting.

Class	Precision	Recall	F1-Score	Support
Ineffective	0.60	0.68	0.64	20
Effective	0.98	0.95	0.96	280
Accuracy			0.93	300
Macro Avg	0.78	0.79	0.79	300
Weighted Avg	0.95	0.93	0.94	300
AUC-ROC	0.89			

## 4 Discussion

### 4.1 Demographic and professional influences on AI perceptions

The study shows that demographic characteristics and professional backgrounds significantly influence perceptions of AI-enhanced pharmacovigilance models in China. Most respondents were hospital staff and corporate employees, with a strong representation of younger professionals, particularly those aged 26–35. This skew toward younger participants likely reflects a generational comfort with technology, contributing to generally positive attitudes toward AI integration (16). The significant participation of females (78%) suggests gender-specific insights, though this imbalance may limit the generalizability of the findings. Additionally, professional experience plays a crucial role, with those having over 10 years of experience more likely to view AI positively, recognizing its potential to improve the accuracy and efficiency of pharmacovigilance, likely due to their familiarity with traditional methods' limitations (17). This is consistent with the findings of Ndagije et al., who identified that healthcare professionals with a better understanding of the limitations of existing systems are more receptive to new technological solutions like AI (18).

### 4.2 Professional role-based perceptions of AI capabilities in pharmacovigilance

The role of AI in pharmacovigilance primarily lies in its ability to enhance monitoring and expedite adverse drug reaction (ADR) processing. Pharmacovigilance center managers showed the strongest support for AI, recognizing its potential to improve efficiency and accuracy in detecting ADRs. In contrast, the varied responses from hospital staff, including notable skepticism, may stem from practical concerns about AI disrupting established clinical workflows, increasing the burden of data entry, or a lack of trust in “black box” algorithms for critical safety assessments (19). This skepticism among hospital staff may be a result of practical challenges in workflow integration and data reliability, which aligns with existing literature on healthcare technology adoption. Corporate employees generally supported AI, reflecting their focus on operational efficiency, while the general public's opinions were more mixed, indicating a need for broader public education on AI's role in healthcare. Overall, while AI demonstrates significant potential, its successful implementation depends on addressing the specific concerns and gaining the acceptance of all stakeholders (20).

### 4.3 Linking pharmacovigilance management challenges with monitoring systems effectiveness

The correlation analysis highlighted the significant link between challenges in pharmacovigilance management, such as inadequate monitoring mechanisms and poor communication, indicating that deficiencies in one area often exacerbate issues in another, reducing overall system effectiveness (21). This interconnectedness underscores the need for a holistic approach to system improvement, where enhancing communication channels and refining monitoring protocols are addressed simultaneously. Additionally, the analysis revealed a slight negative correlation between confidence in the system's capability and the presence of these issues, suggesting that stakeholders who perceive gaps in communication and monitoring are less confident in the system's ability to manage drug safety effectively (22). Addressing these core deficiencies is crucial not only for improving the technical aspects of pharmacovigilance but also for restoring and boosting stakeholder confidence. Prioritizing improvements in these areas could lead to more sustainable enhancements in the pharmacovigilance framework, fostering a more robust and reliable system for managing medication safety (23).

### 4.4 Key factors shaping attitudes towards AI in pharmacovigilance

Regression analysis revealed that professional role and experience are key determinants of attitudes toward AI in pharmacovigilance. Hospital staff and individuals with over 10 years of experience showed a more favorable view of AI's potential, likely due to their familiarity with the limitations of traditional methods and exposure to technological advancements (24). However, the analysis also suggested that other unmeasured factors, such as individual exposure to AI technologies, hands-on experience with AI tools, organizational culture, and the perceived ease of AI integration, could significantly influence perceptions (25). The complexity of these influences highlights the need for further research to identify these additional variables. Understanding how these factors interact with professional roles and experience levels could provide deeper insights into the barriers and facilitators of AI adoption in pharmacovigilance, guiding the design of targeted interventions to improve AI acceptance and integration across various professional groups within the healthcare system (26).

### 4.5 The impact of AI and information systems on the effectiveness of pharmacovigilance

Logistic regression and path analysis highlighted the significant impact of both AI and information systems on the effectiveness of pharmacovigilance management. AI's ability to rapidly process adverse drug reaction (ADR) reports was seen as valuable for efficient monitoring, but the perceived effectiveness of information systems played an even more crucial role in evaluating the overall pharmacovigilance model (27). This suggests that while AI enhances specific tasks, robust information systems are essential for integrating AI-driven insights into an effective management strategy (28). This finding corroborates research by Liang et al. (29) who emphasize that AI tools must be built upon a robust and structured data foundation provided by effective information systems (29). These findings underscore the need for a balanced approach that combines AI's capabilities with strong, well-integrated information systems (30). Such systems are vital for seamless data flow, improved information accessibility, and coordinated responses to drug safety issues. By optimizing the synergy between AI and information systems, pharmacovigilance processes can be significantly enhanced, leading to more reliable and effective medication safety management on a national scale (31).

### 4.6 How AI and information systems empower the “One Body, Two Wings” framework

This research provides a clear model for how technology can empower the “One Body, Two Wings” framework. AI's capacity for rapid ADR processing and enhanced monitoring directly strengthens the capabilities of the pharmacovigilance institutions, which form the “Body” of the system (32). However, our analysis decisively shows that the “Wings”—healthcare institutions and marketing authorization holders—can only function effectively in concert with the body if supported by robust information systems (33). These systems act as the essential connective tissue, ensuring that data flows seamlessly and that AI-driven insights are translated into coordinated action across the entire framework. Therefore, the success of the model is not merely dependent on the intelligence of the “Body” but on the strength and reliability of the technological infrastructure that binds it to its “Wings” (34).

### 4.7 Insights from factor analysis on pharmacovigilance management perceptions

The exploratory factor analysis (EFA) provided statistical validation for three underlying dimensions of pharmacovigilance management perceptions, reinforcing the key themes discussed previously. The first factor highlighted the importance of organizational structure and process efficiency, with strong loadings on city-level frameworks and streamlined management processes, reflecting the value placed on a well-managed pharmacovigilance infrastructure (35). The second factor emphasized technology integration, particularly AI, showing high loadings for its role in enhancing monitoring and expediting adverse drug reaction (ADR) processing, aligning with the broader trend of using AI to improve accuracy, efficiency, and responsiveness in drug safety (36). The third factor underscored the critical role of information systems in coordinated management, with strong loadings indicating their importance in data management, communication, and decision-making. These findings suggest that the success of pharmacovigilance initiatives relies heavily on robust information systems that support AI applications and overall system operations (37).

### 4.8 Implications of decision tree and random forest analyses

The machine learning analyses provide data-driven support for the importance of technological infrastructure in pharmacovigilance. After addressing the inherent class imbalance in survey responses through robust methods (SMOTE and class weighting), both the decision tree and random forest models demonstrated that system effectiveness is a primary predictor of positive outcomes (38). The feature importance analysis from the random forest model is particularly revealing; the high and stable importance score for “Information Systems Effectiveness” (0.53 ± 0.05) suggests that stakeholders perceive a functional IT backbone as the most critical component for the success of the “One Body, Two Wings” framework (39). While AI capabilities in monitoring and ADR processing are significant contributors, their value appears to be maximized when they are integrated into an already effective information system. This implies that policy and investment should prioritize foundational IT infrastructure before or alongside the implementation of advanced AI tools. The models' improved, albeit imperfect, ability to predict “ineffective” cases suggests that while technology is a key factor, organizational or human factors not captured in the current feature set may also play a role in instances of system failure (40).

### 4.9 Recommendations for practice and policy

To translate these findings into practice, a two-pronged approach is essential. Firstly, “targeted education” must be tailored to stakeholder needs: this includes hands-on workshops for hospital staff focusing on AI's integration into clinical workflows and data security, alongside public-facing campaigns to build trust and awareness of AI's role in medication safety (41). Secondly, 'sustained investment' should be directed towards critical infrastructure: primarily, developing standardized Application Programming Interfaces (APIs) to ensure seamless data exchange between hospital EHR systems and national pharmacovigilance databases, and establishing a national-level AI platform for advanced signal detection that integrates multi-source data (42). These concrete steps are vital for translating the potential of AI into tangible improvements in the national pharmacovigilance system (43).

### 4.10 Limitations and future research directions

This study identified several areas where further research is needed. The demographic skew towards younger, female respondents, with females comprising 78% of the sample, suggests a potential limitation in the generalizability of the findings. This imbalance may reflect the gender distribution in certain professional roles within healthcare, but it is a key consideration for interpreting the results. Additionally, the influence of unmeasured factors on AI perceptions indicates that there is room for further exploration into what drives these attitudes. Longitudinal studies could provide insights into how perceptions of AI and information systems in pharmacovigilance evolve over time, particularly as these technologies become more integrated into daily practices.

## 5 Conclusion

This study provides a comprehensive evaluation of AI-enhanced “One Body, Two Wings” pharmacovigilance models in China, highlighting the crucial and synergistic roles of artificial intelligence (AI) and robust information systems. The findings show that while AI improves the efficiency and accuracy of adverse drug reaction (ADR) monitoring, its success relies on a foundational, well-structured information system. The perceived effectiveness of these systems was a stronger and more stable predictor of positive attitudes than AI factors alone, underscoring AI's role as a powerful complement rather than a standalone solution. The study also found that professional roles and experience significantly influence AI acceptance, with insights relevant to global pharmacovigilance strategies. The research concludes that sustained investment in technological infrastructure, particularly in strengthening core information systems, is the key prerequisite for effectively leveraging AI to enhance medication safety management in China and beyond.

## Data Availability

The original contributions presented in the study are included in the article/Supplementary Material, further inquiries can be directed to the corresponding author/s.
