# Supplementary material for: Evaluating the effectiveness of AI-enhanced “One Body, Two Wings” pharmacovigilance models in China: a nationwide survey on medication safety and risk management
Source: Front Health Serv. 2025 Oct 8;5:1655726. doi: 10.3389/frhs.2025.1655726 (PMC12540474; doi:10.3389/frhs.2025.1655726)
Supplement: Supplementary file 1 [file Datasheet1.pdf]

Questionnaire: Constructing a Coordinated Work Model with Two-Wing Integration for Medication Safety Risk Prevention and Control System

Introduction: This questionnaire aims to investigate the current issues in pharmacovigilance management, explore the effectiveness of a coordinated management model based on the integrated two-wing pharmacovigilance framework, and introduce Artificial Intelligence (AI) technology to improve the efficiency and accuracy of pharmacovigilance work. Your responses will contribute to the construction of a medication safety risk prevention and control system. Thank you for your cooperation!
Target Respondents: Pharmacovigilance center managers, hospital staff, corporate employees, the public, etc.

Part I: Basic Information

1. **Your role:**
    - o Pharmacovigilance Center Manager
    - o Hospital Staff
    - o Corporate Employee
    - o Public
2. **Your age:**
    - o 18-25
    - o 26-35
    - o 36-45
    - o 46-60
    - o Above 60
3. **Your gender:**
    - o Male
    - o Female
    - o Other
4. **Years of work experience:**
    - o 1-3 years
    - o 4-7 years
    - o 8-10 years
    - o More than 10 years

Part II: Innovative Coordinated Management Model of the Integrated Two-Wing Pharmacovigilance Framework

1. **What do you think are the main issues in current pharmacovigilance management?** (Multiple choices)
    - o Poor communication
    - o Inadequate monitoring and warning mechanisms
    - o Unsatisfactory follow-up measures
    - o Incomplete regulations and policies
    - o Other (please specify) ___________

2. **How familiar are you with the integrated two-wing coordinated pharmacovigilance management model?**
    - Very familiar
    - Quite familiar
    - Somewhat familiar
    - Not very familiar
    - Not familiar at all
3. **Do you believe that the integrated two-wing coordinated pharmacovigilance management model can effectively enhance medication safety management?**
    - Very effective
    - Quite effective
    - Moderately effective
    - Not very effective
    - Ineffective
4. **Which aspects do you think need the most improvement when implementing the integrated two-wing coordinated pharmacovigilance management model?** (Multiple choices)
    - Organizational structure
    - Work process
    - Information system support
    - Regulations and policies
    - Other (please specify) ___________

Part III: Monitoring, Warning, and Follow-Up of Key Medication Safety Risk Events

1. **What do you think are the main issues in current medication safety risk event monitoring?** (Multiple choices)
    - Inadequate monitoring methods
    - Delayed data collection
    - Inaccurate information analysis
    - Other (please specify) ___________
2. **Do you believe that the existing monitoring system can timely detect potential medication risks?**
    - Fully capable
    - Mostly capable
    - Moderately capable
    - Mostly incapable
    - Completely incapable
3. **How do you view the role of the warning mechanism in medication safety management?**
    - Very important
    - Quite important
    - Moderately important

    - Not very important
    - Not important
4. **Have you ever participated in the follow-up treatment of medication safety risk events?**
    - Yes
    - No
5. **If you have participated, what do you think are the shortcomings of the existing follow-up measures?** (Multiple choices)
    - Slow response time
    - Difficult communication and coordination
    - Inappropriate handling measures
    - Insufficient resources
    - Other (please specify) ___________

Part IV: Expected Goals

Please rate the following statements using a Likert scale (1 = Strongly Disagree, 2 = Disagree, 3 = Neutral, 4 = Agree, 5 = Strongly Agree):

1. **I believe that establishing a city-level organizational structure for pharmacovigilance work is very important.**
    - 1
    - 2
    - 3
    - 4
    - 5
2. **I believe that streamlining the main process management of the integrated two-wing pharmacovigilance work at the city level can improve work efficiency.**
    - 1
    - 2
    - 3
    - 4
    - 5
3. **I believe that conducting coordinated pharmacovigilance management and scientific prevention of major medication risk events based on information systems is effective.**
    - 1
    - 2
    - 3
    - 4
    - 5

Part V: Core Content

Please rate the following statements using a Likert scale (1 = Strongly Disagree, 2 = Disagree, 3 = Neutral, 4 = Agree, 5 = Strongly Agree):

1. **I have participated in the research on the organizational structure of coordinated pharmacovigilance management.**
   - 1
   - 2
   - 3
   - 4
   - 5
2. **I believe that streamlining the main work processes of the integrated two-wing pharmacovigilance and establishing relevant regulations are very important.**
   - 1
   - 2
   - 3
   - 4
   - 5
3. **I believe that applying information systems can significantly enhance the effectiveness of coordinated pharmacovigilance management and medication risk prevention.**
   - 1
   - 2
   - 3
   - 4
   - 5

Part VI: Key Challenges

1. **What do you think are the main difficulties in establishing an effective integrated two-wing coordinated management mechanism?** (Multiple choices)
   - Difficulties in organizational coordination
   - Uneven resource distribution
   - Insufficient information sharing
   - Incomplete regulations and policies
   - Other (please specify) ___________
2. **What do you think are the challenges in establishing convenient and effective management processes and regulations for pharmacovigilance information communication?** (Multiple choices)
   - Insufficient technical support
   - Inadequate staff training
   - Information security concerns
   - Other (please specify) ___________
3. **Do you believe that using information technology tools to support coordinated pharmacovigilance management and medication risk prevention work helps improve work efficiency?**
   - Strongly agree

- o Agree
- o Neutral
- o Disagree
- o Strongly disagree

Part VII: Application of Artificial Intelligence in Pharmacovigilance Work

Please rate the following statements using a Likert scale (1 = Strongly Disagree, 2 = Disagree, 3 = Neutral, 4 = Agree, 5 = Strongly Agree):

1. **I believe that Artificial Intelligence (AI) can significantly enhance the monitoring and warning capabilities of pharmacovigilance.**
   - o 1
   - o 2
   - o 3
   - o 4
   - o 5
2. **I believe that AI can help process adverse drug reaction reports more quickly and accurately.**
   - o 1
   - o 2
   - o 3
   - o 4
   - o 5
3. **What do you think are the main challenges in applying AI technology in pharmacovigilance?** (Multiple choices)
   - o Insufficient data quality and quantity
   - o Lack of professional technical personnel
   - o Unclear regulatory policies
   - o High costs
   - o Other (please specify) ____________

Part VIII: Additional Questions

1. **Do you have any suggestions for the implementation of the integrated two-wing coordinated pharmacovigilance management model?**

   ____
2. **What do you think is the most critical aspect that needs improvement in current pharmacovigilance work?**

   ____

Let me know if you need further assistance!
